# Radial Nerve Palsy Caused by Desmoid-Type Fibromatosis: A Case Report and Review of the Literature

**DOI:** 10.7759/cureus.65008

**Published:** 2024-07-20

**Authors:** Ryuta Iwanaga, Atsushi Mihara, Takashi Sakai, Keiichi Muramatsu, Takahiro Hashimoto

**Affiliations:** 1 Department of Orthopedic Surgery, Yamaguchi University Graduate School of Medicine, Ube, JPN; 2 Department of Hand and Microsurgery, Nagato General Hospital, Nagato, JPN; 3 Department of Orthopedic Surgery, Ubekosan Central Hospital, Ube, JPN

**Keywords:** drop finger, microsurgery, upper extremity, fibromatosis, radial nerve palsy

## Abstract

Radial nerve palsy (RNP) is classified as traumatic, non-traumatic, or iatrogenic. The most frequent etiologic agent is the fracture of the humerus of the shaftand distal. We experienced a case of RNP caused by desmoid-type fibromatosis around the radial nerve. The RNP caused by desmoid-type fibromatosis has not been reported in the literature. We present this case here with a review of the RNP literature. The patient is a 16-year-old female, right-hand dominant, who became aware of the difficulty in extending her right little finger without any triggers five months ago. She was also aware of the difficulty in extending the ring finger, and her symptoms gradually worsened. She was referred to our hospital after consulting a home doctor. MRI of the elbow showed a high-intensity occupying lesion on T2-weighted images (T2WI) slightly proximal to the elbow joint. Ultrasonography (US) showed a partial nerve constriction and radial nerve enlargement on the distal side of the constriction. The approach was made from the posterior lateral side of the distal upper arm, and the radial nerve was exposed. There was a 1 cm white tissue strongly adherent on the radial nerve, which was compressing the radial nerve, and it was resected piece by piece. After the resection, the radial nerve was indented. The pathological diagnosis of the resected tissue was fibromatosis. Gradually, she was able to extend her fingers after the surgery and recovered completely in six months.

## Introduction

Radial nerve palsy (RNP) is classified as traumatic, non-traumatic, or iatrogenic [1-3]. The most frequent etiologic agent is the fracture of the humerus of the shaft and distal [1]. In some cases, such as Saturday night syndrome, RNP is caused by transient compression [4]. The symptoms of RNP are drop hand or finger, and the patient is unable to grasp objects and has difficulty in daily life. However, the symptoms usually recovered with only observation in some months. We experienced a case of RNP caused by desmoid-type fibromatosis around the radial nerve. Desmoid-type fibromatosis is a soft-tissue tumor characterized by a tendency to recur locally, whereas metastasis is rare. It can occur at any site, including the extremities, trunk, and abdominal cavity. It accounts for about 3% of all soft-tissue tumors and is reported to occur in two to four million people per year. It most commonly occurs in people between the ages of 10 and 40, and there is no difference between males and females. Non-traumatic RNP can be caused by the tumor, hourglass-like constriction, or compression by the lateral head of the triceps brachii muscle; however, RNP caused by desmoid-type fibromatosis has not been reported in the literature [2,5,6]. We present this case here with a review of the RNP literature.

## Case presentation

**Figure 1:**
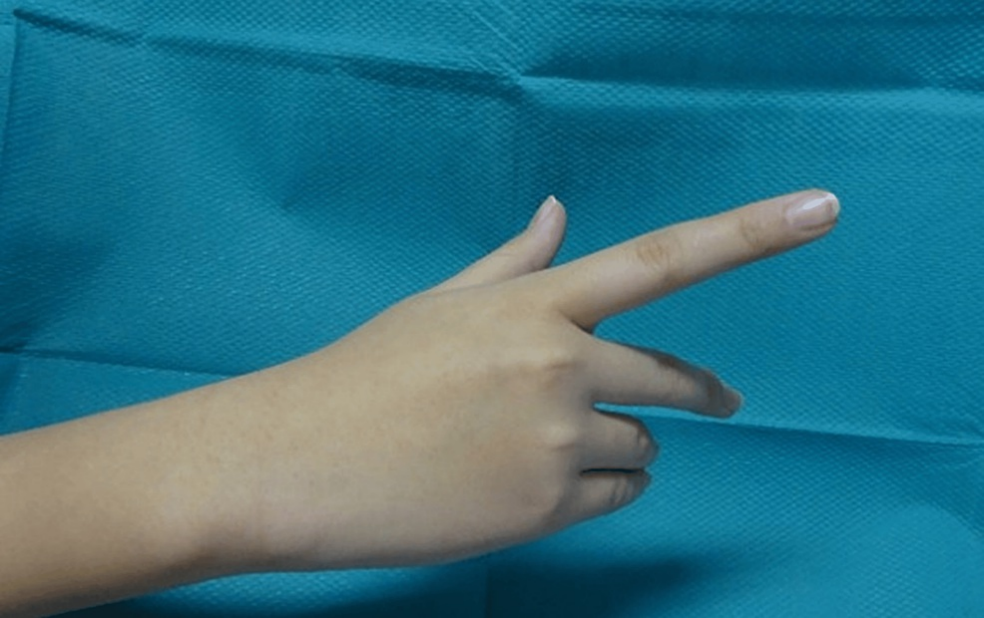
A picture of the patient's right hand at the time of the initial examination is shown. The radial nerve is incompletely palsy with ulnar predominance.

The patient is a 16-year-old female, right-hand dominant, who became aware of the difficulty in extending her right little finger without any triggers five months ago. No history of trauma. She was also aware of the difficulty in extending the ring finger, and her symptoms gradually worsened. She was referred to our hospital after consulting a home doctor. When she came to our hospital, her wrist dorsiflexion MMT was good, the extension of the MP joints of the thumb and index finger was good, the extension of the MP joints of the middle finger was poor, and the extension of the ring finger and little finger was trace (Figure 1). There was no sensory disturbance in the area of the radial nerve. There was no obvious mass touching the right upper extremity, no tenderness, and no Tinel’s sign.

**Figure 2:**
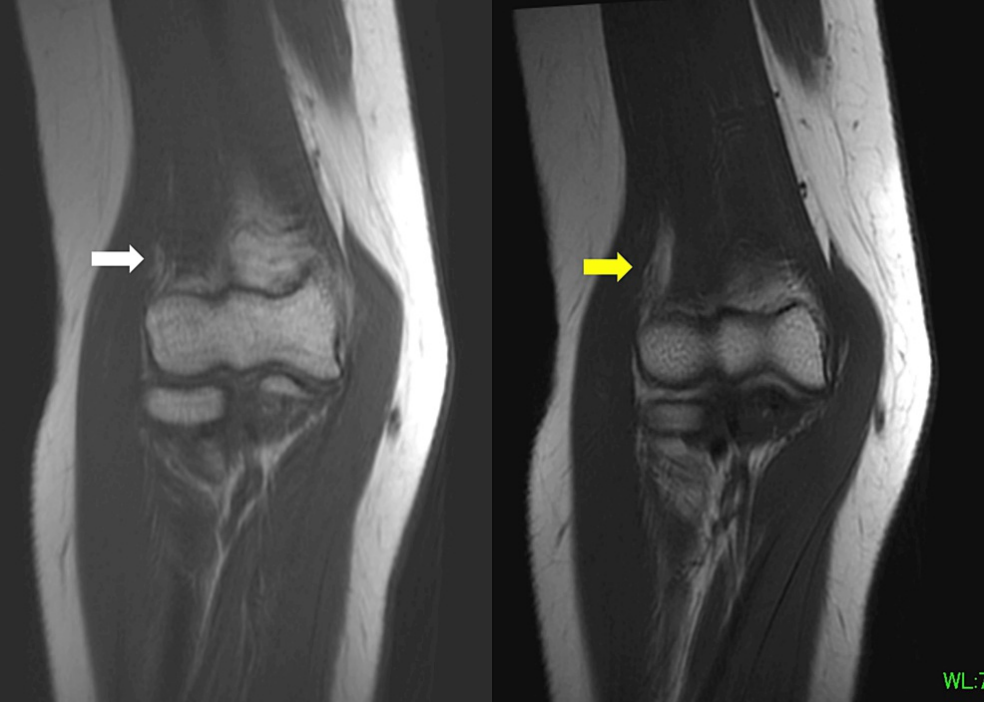
Sagittal view of preoperative MRI. Left side: T1WI. Iso- and high-intensity occupying lesions (white arrow) are seen. Right side: T2WI. High-intensity occupying lesions (yellow arrow) are seen. T2WI, T2-weighted image; T1WI, T1-weighted image

**Figure 3:**
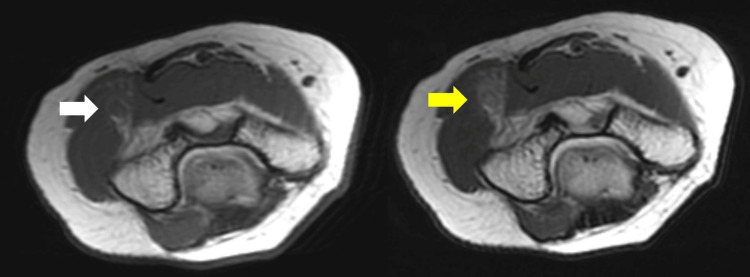
Axial view of preoperative MRI. Left side: T1WI. Iso- and high-intensity occupying lesions (white arrow) are seen. Right side: T2WI. High-intensity occupying lesions (yellow arrow) are seen. T2WI, T2-weighted image; T1WI, T1-weighted image

**Figure 4:**
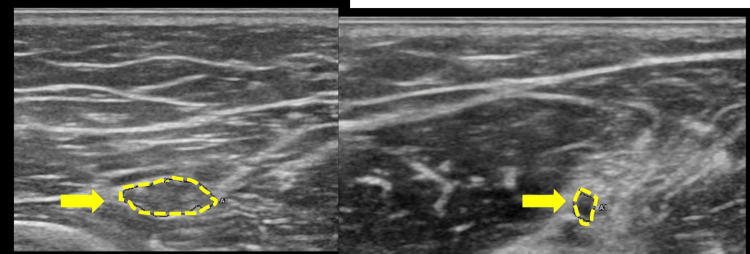
US showing radial nerve (yellow arrows). The radial nerve is enlarged (left side) and its proximal side is narrowed (right side). There is no obvious space-occupying lesion. US, ultrasonography

MRI of the elbow (Figures 2, 3) showed a high-intensity occupying lesion on T2-weighted images (T2WI) slightly proximal to the elbow joint. Ultrasonography (US) showed a partial nerve constriction and radial nerve enlargement on the distal side of the constriction (Figure 4). MRI of the cervical spine showed no abnormalities. We also tried to measure the nerve conduction velocity, but only CMAPS of EIP was derived, and the amplitude was decreased to 0.23 mV. Therefore, we diagnosed the patient with RNP caused by schwannoma, neurofibroma, and neuritis, and decided to perform neurolysis. If the RNP did not recover or worsen after the neurolysis, we planned to perform a secondary tendon transfer.

**Figure 5:**
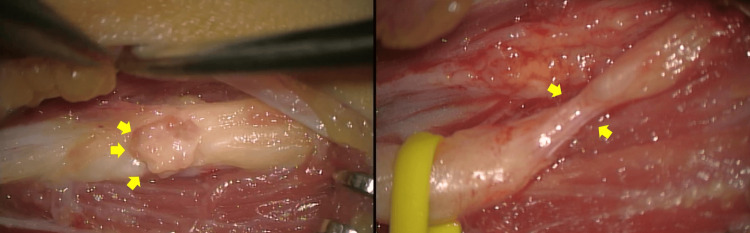
Intraoperative photographs. Left side: 1 cm tumor tissue (yellow arrow) adherent to the radial nerve is seen. Right side: After tumor resection, the radial nerve is compressed and narrowed (yellow arrow).

**Figure 6:**
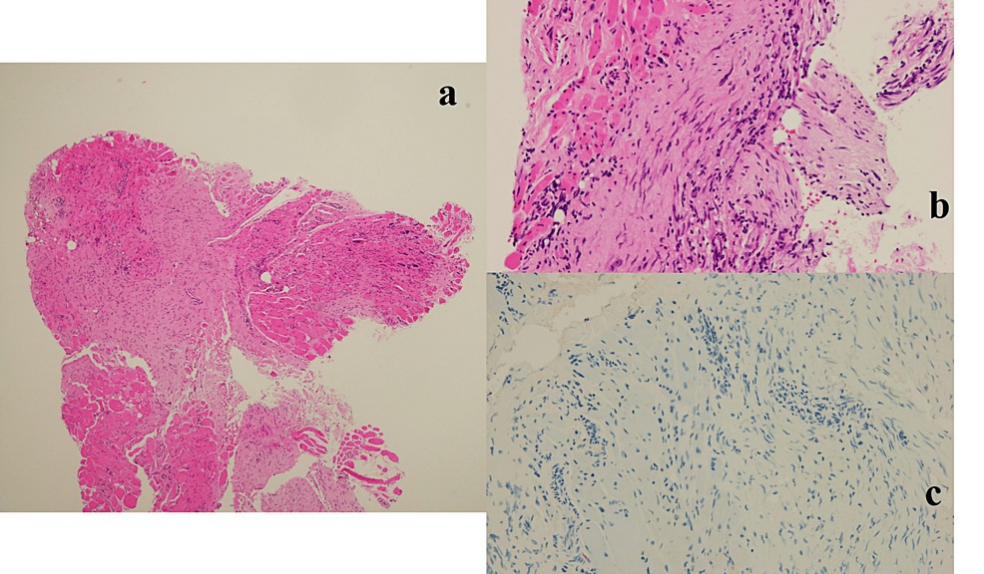
Pathological findings of the lesion are shown in a, b, and c. a) There is a hyperplasia of fibroblasts, extending into the area between the rhabdoid muscles; b) There is no evidence of nuclear atypia; c) S-100 is not expressed.

The approach was made from the posterior lateral side of the distal upper arm, and the radial nerve was exposed. There was a 1 cm white tissue strongly adherent on the radial nerve, which was compressing the radial nerve, and it was resected piece by piece (Figure 5; left side). The tumor was outside the epineurium. The radial nerve was so thinned by the tumor that there was no space for an incision inside the nerve. The procedure was performed under a microscope. After the resection, the radial nerve was indented (Figure 5; right side). The pathological diagnosis of the resected tissue was fibromatosis (Figure 6). Gradually, she was able to extend her fingers after the surgery and recovered completely in six months. Three years later, there has been no local recurrence of tumor or relapse of symptoms.

## Discussion

The review of RNP

**Figure 7:**
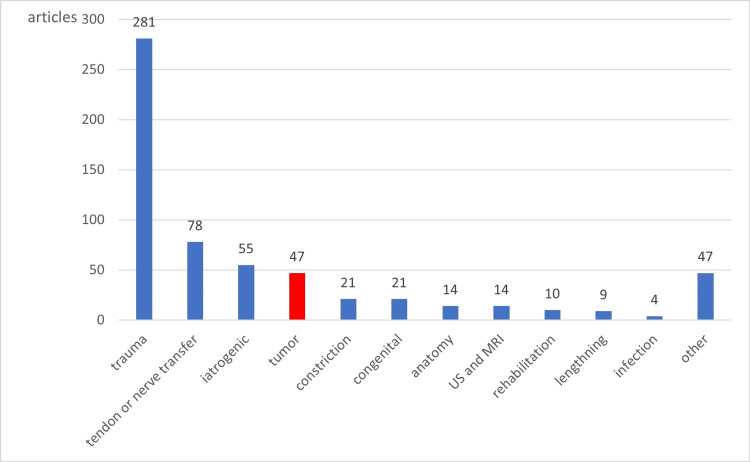
721 articles about RNP. RNP, radial nerve palsy

Pubmed is used to search for articles published between January 1995 and March 2021 using the keywords "radial nerve," "palsy," "constriction," "torsion," and "posterior interosseous nerve.” A total of 721 articles are searched. One hundred twenty articles were excluded after reading the titles and abstracts as not related to RNP. The largest number of articles is related to trauma (281 articles), and 47 articles are related to tumors (Figure 7). Fewer than three articles related to RNP were classified as other, resulting in 47 articles.

**Figure 8:**
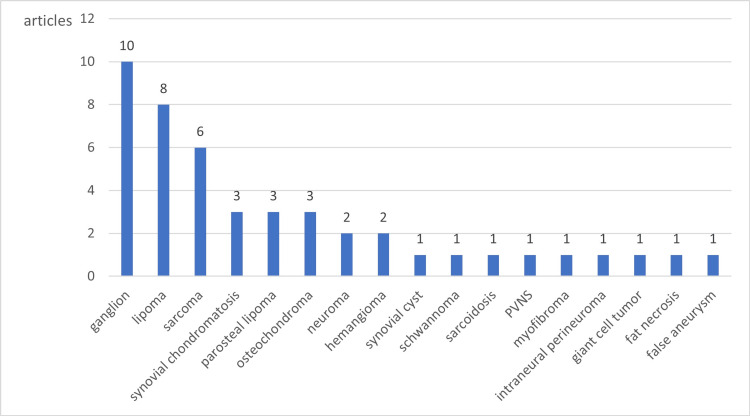
RNP caused by tumor. RNP, radial nerve palsy

The articles related to tumors are reviewed in detail. The largest number of articles (10) is related to ganglion (Figure 8). Most reports of RNP due to bone and soft tissue tumors were soft tissue tumors, and the only bone tumor reported was osteochondroma. There are no articles related to desmoid-type fibromatosis, and this is the first report of our case.

The diagnosis of desmoid-type fibromatosis

According to the WHO classification (2020), fibromatosis is benign and intermediate tumor classified as fibromatosis coli, juvenile hyaline fibromatosis, inclusion body fibromatosis, palmer fibromatosis, and desmoid-type fibromatosis. Fibromatosis coli, inclusion body fibromatosis, and palmer fibromatosis are excluded because of their different sites of origin from this case. Juvenile hyaline fibromatosis is a type of fibromatosis in which hyaline is deposited in the tissues, and the pathological image is different from that of our case. Desmoid-type fibromatosis is consistent with the pathological image and is appropriate because it can occur in the muscle. There have been five reports of neuropathy caused by desmoid-type fibromatosis, but no reports of occurrence in extremities [7,8].

For the following reasons, our case is diagnosed as desmoid-type fibromatosis. The S-100 protein is negative, and it is not considered to be a neurofibroma. The absence of pain before paralysis and the obvious adhesions around the nerve make it unlikely to be an hourglass-like constriction. Nodular fasciitis is usually a self-limited disease, occurring subcutaneously. Nodular fasciitis is also a candidate as a fibrous tumor, but it is excluded because the patient's symptoms did not improve after five months, and instead worsened. There was no history of trauma and no scarring.

Desmoid-type fibromatosis has been reported to have T1WI iso-high in 83% and T2WI low in 62% [9,10]. On preoperative MRI, there is an occupying lesion with iso-intensity on T1WI and high-intensity on T2WI, which indicates tumor or tumor-induced inflammation and scarring.

Why is it no longer possible to extend mainly the little finger and ring finger?

Why is it no longer possible to extend mainly the little finger and ring finger? The cause is unknown to our best knowledge. Tamura et al. reported the intraneural localization atlas of the radial nerve [11]. The radial nerve clearly separates into motor and sensory nerves 3 cm proximally from the line connecting the medial and lateral epicondyle of the humerus. In our case, the tumor is located on the 3 cm line proximally, which may have caused motor-dominant paralysis. There may also be an abnormality in the running of sensory nerves, as pointed out by Day et al. [12]. However, the detailed funicular pattern of the motor nerves is unknown. A detailed intraneural localization atlas has been reported for the median nerve, whereas none has been reported for the posterior interosseous nerve [13].

The future of our case

Desmoid-type fibromatosis sometimes has a recurrence rate of 80-90% with marginal resection. In our case, we had to perform a marginal resection because the tumor was adherent to the muscles and nerves. In addition, the tumor did not invade into the radial nerve, was compressible, and could be totally resected under the microscope. Therefore, the possibility of recurrence is high, and careful follow-up is required. If recurrence occurs, treatment is done with COX-2 inhibitors, Tranilast, tamoxifen, MTX, imatinib, etc. [14-17]. Wait and see is one method until nerve palsy appears, as this is a self-limited disease.

## Conclusions

We report a case of the RNP caused by desmoid-type fibromatosis. After resection under a microscope, the patient recovered in about six months after surgery. Careful follow-up is required to monitor the patient for recurrence.
